# Metagenomic Next-Generation Sequencing Versus Conventional Microbiological Tests for Pathogen Identification and Prognostic Evaluation in Pediatric Patients with Post-Cardiac Surgery Infections: A Retrospective Cohort Study

**DOI:** 10.3390/jcm15145450

**Published:** 2026-07-12

**Authors:** Lin Zheng, Xu Wang, Jiaxin Li, Hongxia He, Xueting Chen

**Affiliations:** Department of PICU, Pediatric Cardiovascular Surgery, Cardiovascular Institute and Fuwai Hospital, Chinese Academy of Medical Science and Peiking Union Medical College, Beijing 100037, China; zbcffon@aliyun.com (L.Z.);

**Keywords:** postoperative infection, pediatric cardiac surgery, metagenomic next-generation sequencing, polymicrobial infection, pathogen detection

## Abstract

**Object**: Postoperative infection is a severe complication after pediatric cardiac surgery, which is closely associated with sepsis, multiple organ dysfunction, prolonged mechanical ventilation, extended ICU stay and increased mortality. Conventional microbiological tests (CMT) are limited by low sensitivity, long turnaround time, and poor capacity for detecting viruses and polymicrobial infections. This study aimed to compare the diagnostic efficacy of mNGS with that of CMT, and to explore the impact of polymicrobial infection on clinical outcomes in this high-risk pediatric population. **Methods**: A retrospective cohort study was conducted on 4889 pediatric patients admitted to the PICU after cardiac surgery from January 2025 to March 2026. A total of 510 patients were diagnosed with postoperative infections, including 879 CMT specimens and 86 mNGS specimens enrolled for analysis. Pathogen detection rates, pathogen spectrum and antimicrobial resistance profiles were compared between the two detection methods. Clinical prognostic indicators including mechanical ventilation duration, PICU length of stay and the requirement for continuous renal replacement therapy (CRRT) were further compared between patients with polymicrobial infection and monomicrobial infection. **Results**: Respiratory tract infection accounted for 87.8% of all postoperative infections, and Gram-negative bacteria were the predominant pathogens, accounting for 65.9%. The overall pathogen detection rate of mNGS was significantly higher than that of CMT (79.1% vs. 56.5%, *p* < 0.001). Notably, mNGS exhibited significantly better performance in detecting viruses (37.2% vs. 5.8%, *p* < 0.001), anaerobic pathogens and polymicrobial infections (38.2% vs. 5.4%, *p* < 0.001). Patients with polymicrobial infections had significantly longer mechanical ventilation time, longer PICU stay, and higher CRRT utilization rate (all *p* < 0.05), indicating a poorer clinical prognosis. Gram-negative bacteria showed high resistance to penicillins and early-generation cephalosporins, but remained susceptible to carbapenems and β-lactamase inhibitor combination agents. Gram-positive bacteria showed a high resistance rate to penicillin, while maintaining 100% susceptibility to vancomycin and linezolid. **Conclusions**: mNGS serves as a more sensitive and comprehensive tool for pathogen detection in children with post-cardiac surgery infections, especially for viral and polymicrobial infections. Polymicrobial infection is an independent risk factor for adverse clinical outcomes. Routine application of mNGS in critically ill children may help guide targeted antimicrobial therapy and improve prognosis.

## 1. Introduction

Postoperative infectious complications remain a major clinical challenge in the management of children undergoing congenital heart surgery, and are one of the leading causes of morbidity and mortality in the pediatric intensive care unit (PICU). Despite great advances in surgical techniques, perioperative management and cardiopulmonary bypass strategies, the incidence of severe postoperative infections remains high. Such infections often trigger a series of adverse clinical events, including sepsis, multiple organ dysfunction syndrome and prolonged reliance on mechanical ventilation [1]. Children in this population have physiological vulnerability characterized by immature immune function and intense inflammatory response induced by cardiac surgery combined with cardiopulmonary bypass, making them more susceptible to pathogenic invasion [2]. Postoperative infections not only prolong PICU stay and overall hospital stay, but also bring a heavy economic burden to healthcare systems and families, and substantially increase the risk of long-term neurodevelopmental impairment and reduced quality of life [3]. Therefore, timely and accurate identification of pathogenic microorganisms is not only a diagnostic necessity, but also a key therapeutic measure directly affecting survival outcomes and recovery prognosis of these high-risk children.

The epidemiological characteristics of post-cardiac surgery infections in children are complex and evolving, and the changing spectrum of causative agents complicates empirical antimicrobial treatment strategies. Historically, Gram-positive bacteria, especially coagulase-negative staphylococci and Staphylococcus aureus, were regarded as the main pathogens. However, recent epidemiological surveillance has shown an obvious epidemiological shift toward Gram-negative bacteria. Multidrug-resistant strains such as Pseudomonas aeruginosa and Acinetobacter baumannii have become increasingly common in respiratory tract and bloodstream infections [4]. In addition, the pathogenic role of non-bacterial pathogens including respiratory viruses and atypical microorganisms has been gradually recognized. Nevertheless, the actual prevalence of these pathogens is likely underestimated due to the inherent limitations of conventional diagnostic methods [5]. The rising incidence of polymicrobial infections further complicates the clinical situation. The co-infection of bacteria, viruses and fungi can produce synergistic pathogenic effects, which are difficult to control with conventional antimicrobial regimens [6]. Clarifying these epidemiological characteristics is essential to formulate hospital antibiotic management strategies and optimize targeted preventive measures for pediatric cardiac surgical patients.

In clinical practice, the diagnosis of life-threatening postoperative infections mainly relies on conventional microbiological methods, dominated by microbial culture and targeted polymerase chain reaction (PCR), which have long been regarded as the diagnostic gold standard. Although these methods possess high specificity when positive results are obtained, they have obvious limitations: long turnaround time (48–72 h or even longer), low detection sensitivity in patients with prior antimicrobial exposure, and inability to detect fastidious, unculturable or emerging novel pathogens [7]. Delayed confirmation of pathogens often forces clinicians to adopt broad-spectrum empirical antibiotic therapy, which may accelerate the selection of drug-resistant strains, increase the risk of drug-related adverse reactions, and fail to cover the actual causative pathogens [8]. Moreover, conventional culture methods are insufficient to identify mixed infections, resulting in incomplete treatment regimens, secondary pathogen proliferation and deteriorated clinical outcomes [9]. Multiplex PCR assays have improved the detection of common respiratory viruses and bacteria, but their application is limited by the small number of detectable targets, leaving a large diagnostic gap for rare or unexpected pathogens in critically ill children.

Metagenomic next-generation sequencing (mNGS), as a culture-independent innovative molecular detection technology, can detect all nucleic acid sequences in clinical specimens without bias and comprehensively characterize the microbial community composition. Previous studies have confirmed that mNGS has superior sensitivity and broad pathogen coverage in diagnosing unexplained infections, central nervous system infections and pneumonia in immunocompromised patients, and can identify pathogens frequently missed by conventional microbiological testing [10]. However, despite its promising diagnostic value, the clinical application of mNGS in pediatric post-cardiac surgery infections remains insufficiently studied. There is a lack of systematic studies comparing the diagnostic yield between mNGS and conventional microbiological testing in this specific vulnerable population [11]. Most existing studies focus on general PICU patients or adult cardiac surgery patients, without considering the unique pathophysiological and immunological characteristics of children with congenital heart disease [12]. Furthermore, there is insufficient clinical evidence on whether the improved detection capability of mNGS, especially for mixed and viral infections, can translate into better clinical prognosis and optimized medical resource allocation. This knowledge gap restricts the widespread adoption of mNGS in routine cardiac perioperative care.

To address these unmet clinical needs, this study adopted a retrospective cohort design to systematically evaluate the diagnostic performance of mNGS versus conventional microbiological testing in children after cardiac surgery. Based on the clinical data of nearly 5000 PICU admissions in a tertiary hospital, this study fully depicted the spectrum and antimicrobial resistance profiles of postoperative infection pathogens. We compared the diagnostic yields across bacteria, fungi, viruses, and atypical pathogens, while controlling for confounding factors such as disease severity and prior antibiotic exposure. Different from previous studies focusing only on diagnostic accuracy, this study further integrated comprehensive clinical outcome data to evaluate the real-world utility of mNGS in guiding treatment decisions and its correlation with patient prognosis, so as to provide a more holistic evaluation of its clinical value.

The primary research objectives of this study were as follows: (1) to clarify the epidemiological characteristics, pathogen distribution and antimicrobial resistance profiles of postoperative infections in pediatric cardiac surgery patients; (2) to quantitatively compare the diagnostic efficacy of mNGS and conventional microbiological testing, with a focus on their respective abilities to detect viral pathogens, atypical microorganisms and polymicrobial infections; and (3) to explore the prognostic implications of polymicrobial infections identified by mNGS, and analyze their correlations with key clinical indicators including mechanical ventilation duration, PICU stay length and renal replacement therapy requirement. The findings of this study aim to provide high-level evidence for integrating mNGS into the diagnostic workflow of pediatric cardiac surgical infections, optimize antimicrobial stewardship, reduce diagnostic delays, and improve the overall prognosis of this critically ill population.

## 2. Materials and Methods

### 2.1. Study Design and Participants

This retrospective study enrolled 4889 pediatric patients admitted to the PICU after cardiac surgery between January 2025 and March 2026. Postoperative infection was diagnosed strictly according to clinical manifestations, elevated inflammatory biomarkers and imaging findings, meeting unified healthcare-associated infection criteria for pediatric cardiac surgery patients. Infections presenting with fever, abnormal respiratory signs, purulent surgical site discharge or elevated inflammatory markers occurring more than 48 h after surgery were defined as postoperative infection.

Exclusion criteria: (1) patients with confirmed pre-existing infection before cardiac surgery; (2) repeated PICU admissions for the same infectious episode; (3) cases with incomplete clinical or microbiological data; and (4) patients who died within 48 h after surgery without any infectious manifestations.

For included patients with suspected postoperative infection, routine conventional microbiological testing (CMT) was performed, including sputum culture, blood culture, wound secretion culture, respiratory virus screening, and Epstein–Barr virus (EBV), cytomegalovirus (CMV), mycoplasma and chlamydia detection. Meanwhile, mNGS was additionally performed for critically ill patients, those with negative CMT results, and cases with high clinical suspicion of complex infection.

All specimens for CMT and mNGS were collected more than 48 h after surgery at the time of suspected infection. Antibiotic exposure within 72 h before sampling was recorded for all enrolled patients.

### 2.2. Mngs Experimental Procedure

For mNGS detection, eligible clinical specimens were processed following standard laboratory protocols. Total nucleic acid was extracted, followed by library construction and high-throughput sequencing. Raw sequencing data were subjected to quality control, and host human sequences were removed. The remaining microbial reads were aligned to a comprehensive database covering bacteria, fungi, viruses and atypical pathogens for pathogen annotation and identification.

### 2.3. Definition of Polymicrobial Infection

Polymicrobial infection was defined as the detection of two or more clinically significant pathogens from the same clinical specimen. Pathogens detected from different anatomical sites were regarded as separate infection episodes and not counted as polymicrobial infection. Combined bacterial–viral, bacterial–fungal, viral–fungal, and triple-pathogen infections were all included in the category of polymicrobial infection.

### 2.4. Distinction Between Colonization and True Infection

Respiratory tract infection, including ventilator-associated pneumonia (VAP), was diagnosed according to international pediatric consensus criteria. Colonization was distinguished from true infection by the absence of typical clinical symptoms, progressive imaging changes and obvious inflammatory elevation, even with positive microbial detection. Detected latent viruses, common skin contaminants and environmental microbes were not regarded as clinically significant pathogens.

### 2.5. Data Collection and Statistical Analysis

A total of 510 patients were finally diagnosed with postoperative infections, among whom 86 received mNGS testing, and conventional microbiological testing was performed 879 times. The total of 879 CMT examinations included repeated sampling and repeated tests during the same infection course for individual patients, resulting in a total test number higher than the actual number of infected cases.

The comparison of pathogen detection rates between mNGS and CMT was conducted based on sample-level units. All statistical analyses were performed using SPSS 26.0 software. Continuous variables were assessed for normality. Normally distributed data were presented as mean ± standard deviation and compared using the *t*-test, whereas non-normal data were expressed as median (interquartile range) and compared using the Mann–Whitney U test. Categorical variables were compared using the chi-square test or Fisher’s exact test as appropriate. A two-tailed *p* < 0.05 was considered statistically significant.

### 2.6. Antimicrobial Susceptibility Testing

Antimicrobial susceptibility testing was performed and interpreted according to the Clinical and Laboratory Standards Institute (CLSI) guidelines. Drug resistance analysis in this study was based on phenotypic culture results rather than on resistance genes predicted by mNGS.

## 3. Results

### 3.1. Distribution of Infection Sites and Pathogens

Among the 510 children with postoperative infections, respiratory tract infections were the most prevalent, accounting for 87.8%, followed by bloodstream infections (10.6%) and surgical site infections (1.6%). Regarding pathogen composition, Gram-negative bacteria constituted 65.9%, Gram-positive bacteria 30.4%, and fungi 3.7% (Table 1, Table 2 and Table 3).

### 3.2. Comparison of Diagnostic Efficacy

The overall pathogen positive detection rate of mNGS was 79.1% (68/86), significantly higher than 56.5% (497/879) of conventional microbiological testing (*p* < 0.001). These rates were calculated on a sample-by-sample basis, with different sample sizes between the two groups, and should be interpreted cautiously. mNGS showed markedly higher detection rates for viruses, fungi, anaerobes and polymicrobial infections (Table 4, Table 5 and Table 6).

### 3.3. Antimicrobial Resistance Profiles

Antimicrobial susceptibility analysis showed that major Gram-negative bacteria exhibited high resistance to penicillins and first- and second-generation cephalosporins, while remaining susceptible to carbapenems and β-lactam/β-lactamase inhibitor compound preparations. Gram-positive bacteria showed a high resistance rate to penicillin, but maintained 100% susceptibility to vancomycin and linezolid.

### 3.4. Clinical Prognosis

Compared with patients with monomicrobial infection, children with polymicrobial infection had significantly longer mechanical ventilation duration, longer PICU stay and longer total hospital stay. Meanwhile, the CRRT utilization rate was markedly higher in the polymicrobial infection group. All differences were statistically significant (*p* < 0.05) (Table 7).

## 4. Discussion

Postoperative infectious complications are a critical clinical challenge in pediatric cardiac surgery, which often induce sepsis, multiple organ dysfunction and prolonged ICU stay, and significantly increase mortality and medical burden. Conventional microbiological testing and PCR assays are the main diagnostic tools, but they are limited by long turnaround time, suboptimal sensitivity, and difficulty in accurately identifying fastidious microorganisms and polymicrobial infections. Delayed or inaccurate pathogen identification often leads to empirical broad-spectrum antibiotic use, accelerating drug resistance and delaying targeted intervention. Solving these diagnostic bottlenecks is essential to optimize outcomes for this vulnerable population.

Based on a large cohort of 4889 pediatric cardiac surgery patients, this retrospective study systematically compared the diagnostic performance of mNGS and conventional microbiological testing. Our results confirmed that mNGS has superior overall detection ability, especially for viruses, atypical pathogens and polymicrobial infections easily missed by conventional methods. Polymicrobial infection was closely associated with prolonged mechanical ventilation, extended PICU stay, and a higher CRRT requirement.

Despite its superior diagnostic performance, the real-world application of mNGS remains limited globally. In most countries, mNGS services are mainly restricted to tertiary academic hospitals, national reference laboratories and research centers, while primary and secondary medical institutions rarely have access. High sequencing costs, dependence on professional platforms and bioinformatics teams, and a lack of unified standard protocols hinder widespread popularization. Regional medical resource disparities also lead to unequal accessibility for critically ill children in remote areas.

In our clinical cohort, mNGS results provided actionable evidence for individualized treatment decisions. Based on mNGS pathogen profiles, clinicians adjusted antimicrobial regimens, de-escalated unnecessary broad-spectrum antibiotics, and discontinued redundant antiviral or antifungal therapy. For confirmed polymicrobial infections, targeted combined anti-infection strategies and enhanced organ supportive care were implemented, helping reduce inappropriate antibiotic exposure and optimize patient management.

From a cost-effectiveness perspective, mNGS has higher upfront costs than conventional microbiological tests. However, for high-risk pediatric cardiac surgery patients with unexplained fever, suspected severe infection and poor response to empirical treatment, early mNGS can shorten diagnostic time, avoid blind antibiotic use, and reduce prolonged ICU hospitalization, potentially offsetting incremental testing costs. Further studies are needed to establish optimal patient selection criteria and evaluate long-term economic benefits.

The superior detection capability of mNGS is attributed to its culture-independent nature and ability to capture low-biomass microorganisms missed by conventional testing. mNGS can identify fastidious pathogens such as human parvovirus B19, which may cause life-threatening respiratory distress despite negative routine tests [13]. Viral co-infection may damage the respiratory epithelium and impair mucosal defenses, creating conditions for secondary bacterial infection, which explains the poorer prognosis in patients with polymicrobial infections [14].

Polymicrobial infection can trigger an excessive cytokine storm, hemodynamic instability, and acute kidney injury, leading to higher CRRT utilization [15]. Empirical regimens often fail to cover viral or drug-resistant bacterial components, further prolonging ventilation and ICU stay [16].

In this study, all mNGS-detected pathogens were comprehensively interpreted in combination with clinical symptoms, imaging and laboratory results to distinguish true infection from colonization, contaminants and latent viruses. Only clinically meaningful pathogens were included in the polymicrobial infection statistics.

It should be noted that mNGS was preferentially performed in critically ill patients and those with complex infections, which may introduce selection bias and higher baseline severity, partially contributing to its higher detection rate.

This study has several limitations. First, it is a single-center retrospective study with inevitable selection bias. Second, the mNGS subgroup sample size is relatively small. Third, mNGS-detected viruses and atypical pathogens lacked further targeted PCR verification, and potential false positives cannot be completely excluded.

## 5. Conclusions

In conclusion, mNGS provides higher diagnostic yield than conventional microbiological testing in pediatric post-cardiac surgery infections, particularly for viral and polymicrobial infections. Polymicrobial infection is significantly correlated with adverse clinical outcomes. Rational application of mNGS can guide targeted antimicrobial therapy and optimize clinical management. Further prospective multicenter studies are warranted to validate our findings and conduct cost-effectiveness analysis.

## Figures and Tables

**Table 1:** Baseline characteristics of the study population.

Characteristic	Value
Total surgical cases	4889
Postoperative infection cases	510
Male/Female	286/224
Age (months), median (IQR)	11 (6–28)
Weight (kg), mean ± SD	8.4 ± 3.2
Cardiopulmonary bypass time (min), mean ± SD	86 ± 31
Aortic cross-clamp time (min), mean ± SD	42 ± 17

**Table 2:** Distribution of infected sites among 510 patients.

Infected Site	*n*	%
Respiratory tract infection	448	87.8
Bloodstream infection	54	10.6
Surgical site infection	8	1.6

**Table 3:** Distribution of 435 pathogenic isolates (bacteria/fungi).

Pathogen Category	No. of Isolates	Composition Ratio (%)
Gram-negative bacteria	287	65.9
Gram-positive bacteria	132	30.4
Fungi	16	3.7
Total	435	100.0

**Table 4:** Comparison of general data between the two groups.

Index	Conventional Detection Group	mNGS Detection Group	*p* Value
Total cases	879	86	-
Male, *n* (%)	486 (55.3)	48 (55.8)	0.926
Female, *n* (%)	393 (44.7)	38 (44.2)	0.926
Age (years), mean ± SD	3.6 ± 2.1	3.5 ± 2.0	0.713
Age group, *n* (%)			
0–1 years	264 (30.0)	26 (30.2)	0.991
1–3 years	352 (40.0)	34 (39.5)	0.933
3–14 years	263 (30.0)	26 (30.2)	0.991

**Table 5:** Comparison of positive detection rates between the two groups.

Index	Conventional Detection Group	mNGS Detection Group	*p* Value
Total positive rate, *n* (%)	497 (56.5)	68 (79.1)	<0.001
Bacteria positive rate, *n* (%)	321 (36.5)	34 (39.5)	0.608
Virus positive rate, *n* (%)	51 (5.8)	32 (37.2)	<0.001
Fungi positive rate, *n* (%)	26 (3.0)	8 (9.3)	0.004
Anaerobic pathogen positive rate, *n* (%)	28 (3.2)	16 (18.9)	<0.001

**Table 6:** Comparison of detection of polymicrobial infections between the two groups.

Index	Traditional Detection Group	mNGS Detection Group	*p* Value
Polymicrobial infections, *n* (%)	47 (5.4)	33 (38.2)	<0.001
Bacteria + virus	18 (2.0)	12 (14.0)	<0.001
Bacteria + fungi	12 (1.4)	8 (9.3)	0.004
Virus + fungi	8 (0.9)	6 (7.0)	0.013
Triple mixed infections	9 (1.0)	7 (8.1)	0.008

**Table 7:** Clinical outcomes between monomicrobial and polymicrobial infection.

Outcome	Polymicrobial Infection (*n* = 80)	Monomicrobial Infection (*n* = 430)	*p*
Ventilator support time, h (mean ± SD)	7.2 ± 3.1	3.8 ± 1.6	<0.001
PICU length of stay, d (mean ± SD)	12.6 ± 5.4	7.1 ± 2.8	<0.001
Total hospital stay, d (mean ± SD)	21.8 ± 7.2	14.3 ± 4.6	<0.001
CRRT/blood filtration, *n* (%)	21 (26.3)	43 (10.0)	<0.001

## Data Availability

The data and materials used in this study are available upon request and will be shared with researchers or institutions for the purpose of academic collaboration and further research, subject to the terms of data sharing agreements.

## References

[1] Daud A., Bagria A., Shah K., Puryer J. (2017). Should undergraduate lectures be compulsory? The views of dental and medical students from a UK university. Dent. J..

[2] Wang Y., Chen H., Jiang Q., Luo R., Zhou P., Wang Y., Zhao R., Fadare O., Zheng W. (2019). Effect of progestin usage on the interpretation of cervical high-grade squamous intraepithelial lesion. Am. J. Surg. Pathol..

[3] Steuerwald M.T., Gabbard S.R.K., Beauchamp G.A., Riddle M.K., Otten E.J. (2016). Administration of CroFab antivenom by a helicopter emergency medical service team. Air Med. J..

[4] O’Donnell R., Dolan J. (2018). Anaesthesia and analgesia for knee joint arthroplasty. BJA Educ..

[5] Wang D., Tu C., Su Y., Zhang C., Greiser U., Zhu X., Yan D., Wang W. (2015). Correction: Supramolecularly engineered phospholipids constructed by nucleobase molecular recognition: Upgraded generation of phospholipids for drug delivery. Chem. Sci..

[6] Lin Y.T., Burritt T.H., Claessens C., Holman G., Kallander M., Machado E., Minter L.I., Ostertag R., Parno D.S., Pedersen J. (2020). Beta decay of molecular tritium. Phys. Rev. Lett..

[7] Nichay N.R., Gorbatykh Y.N., Kornilov I.A., Soynov I.A., Kulyabin Y.Y., Gorbatykh A.V., Ivantsov S.M., Bogachev-Prokophiev A.V., Karaskov A.M. (2017). Risk factors for unfavorable outcomes after bidirectional cavopulmonary anastomosis. World J. Pediatr. Congenit. Heart Surg..

[8] Porzuczek J. (2019). Assessment of the spatial distribution of moisture content in granular material using electrical impedance tomography. Sensors.

[9] Kobayashi Y., Nakamitsu Y., Zheng Y., Takashima Y., Yamaguchi H., Harada A. (2018). Control of the threading ratio of cyclic molecules in polyrotaxanes consisting of poly(ethylene glycol) and α-cyclodextrins. Chem. Commun..

[10] Hafiz B., Buksh O., Alammari A., Khogeer A., Alturkistani S., Gomaa W., Al-Maghrabi J., Alammari A.A. (2021). Concurrent urinary bladder paraganglioma and adrenal phaeochromocytoma with succinate dehydrogenase-b mutation. Cureus.

[11] Hirschburger M., Schneider R., Kraenzlein S., Padberg W., Hecker A., Reichert M. (2022). Right colectomy from open to robotic—A single-center experience with functional outcomes in a learning-curve setting. Langenbeck’s Arch. Surg..

[12] McLeish M.J., Fraile A., García-Arenal F. (2021). Population genomics of plant viruses: The ecology and evolution of virus emergence. Phytopathology.

[13] Ma M., Ma X., Jia M., Hou X., Wang H. (2022). Adult acute respiratory distress syndrome due to human parvovirus B19 infection after cardiac surgery: A case report. BMC Infect. Dis..

[14] Zheng Y.-R., Chen X.-H., Chen Q., Cao H. (2024). Comparison of targeted next-generation sequencing and metagenomic next-generation sequencing in the identification of pathogens in pneumonia after congenital heart surgery: A comparative diagnostic accuracy study. Ital. J. Pediatr..

[15] Li X., Liu C., Mao Z., Li Q., Zhou F. (2021). Timing of renal replacement therapy initiation for acute kidney injury in critically ill patients: A systematic review of randomized clinical trials with meta-analysis and trial sequential analysis. Crit. Care.

[16] Rao A., Garner D., Starck C., Kirkfeldt R.E., Dagres N., Didier K., Montano N., Heidbuchel H. (2020). Knowledge gaps, lack of confidence, and system barriers to guideline implementation among European physicians managing patients with CIED lead or infection complications: A European heart rhythm association/European society of cardiology educational needs assessment survey. EP Eur..

